# Treatment of Sialolithiasis: What Has Changed? An Update of the Treatment Algorithms and a Review of the Literature

**DOI:** 10.3390/jcm11010231

**Published:** 2021-12-31

**Authors:** Michael Koch, Konstantinos Mantsopoulos, Sarina Müller, Matti Sievert, Heinrich Iro

**Affiliations:** Department of Otorhinolaryngology and Head and Neck Surgery, University of Erlangen–Nuremberg, 91054 Erlangen, Germany; Konstantinos.mantsopoulos@uk-erlangen.de (K.M.); sarina.mueller@uk-erlangen.de (S.M.); Matti.sievert@uk-erlangen.de (M.S.); heinrich.iro@uk-erlangen.de (H.I.)

**Keywords:** salivary gland, sialolithiasis, treatment, sialendoscopy, intraductal lithotripsy, transoral duct surgery, extracorporeal lithotripsy, combined treatment

## Abstract

Treatment for sialolithiasis has undergone significant changes since the 1990s. Following the development of new minimally invasive and gland-preserving treatment modalities, a 40–50% rate of gland resection was reduced to less than 5%. Extracorporeal shock-wave lithotripsy (ESWL), refinement and extension of methods of transoral duct surgery (TDS), and in particular diagnostic and interventional sialendoscopy (intSE) are substantial parts of the new treatment regimen. It has also become evident that combining the different treatment modalities further increases the effectiveness of therapy, as has been especially evident with the combined endoscopic–transcutaneous approach. In the wake of these remarkable developments, a treatment algorithm was published in 2009 including all the known relevant therapeutic tools. However, new developments have also taken place during the last 10 years. Intraductal shock-wave lithotripsy (ISWL) has led to remarkable improvements thanks to the introduction of new devices, instruments, materials, and techniques, after earlier applications had not been sufficiently effective. Techniques involving combined approaches have been refined and modified. TDS methods have been modified through the introduction of sialendoscopy-assisted TDS in submandibular stones and a retropapillary approach for distal parotid sialolithiasis. Recent trends have revealed a potential for significant changes in therapeutic strategies for both major salivary glands. For the submandibular gland, ISWL has replaced ESWL and TDS to some extent. For parotid stones, ISWL and modifications of TDS have led to reduced use of ESWL and the combined transcutaneous–sialendoscopic approach. To illustrate these changes, we are here providing an updated treatment algorithm, including tried and tested techniques as well as promising new treatment modalities. Prognostic factors (e.g., the size or location of the stones), which are well recognized as having a strong impact on the prognosis, are taken into account and supplemented by additional factors associated with the new applications (e.g., the visibility or accessibility of the stones relative to the anatomy of the duct system).

## 1. Introduction

Sialolithiasis represents 40–60% and 70–85% of all obstructive diseases in the submandibular and parotid glands, respectively [1]. Up to 80% of all stones are located in the submandibular gland [2]. Following the further development of minimally invasive techniques such as transoral duct surgery (TDS) [3,4,5] and the introduction of new minimally invasive techniques such as extracorporeal shock-wave lithotripsy (ESWL) [6,7,8,9,10] and interventional sialendoscopy (intSE) [11,12,13,14] between the 1990s and the first decade of the 2000s, the gland resection rate has been substantially reduced—to less than 10% or even less than 5% [15,16]. In particular, sialendoscopy-assisted techniques have been reported to contribute to a reduction in symptoms [17]. ESWL was then partly replaced by the introduction of combined approaches, particularly for parotid stones [18,19,20].

All of these minimally invasive techniques became established as part of the state of the art for treating sialolithiasis and were regarded as forming a substantial part of the published minimally invasive treatment regimens [5,16,21,22].

The size, location, mobility, and shape of the stone were recognized as prognostic factors influencing the treatment results [23] and have all been taken into account in the new treatment algorithms, except for the shape of the stone. All of these parameters can be visualized by using high resolution ultrasound, which proved to be a fast, effective and well suited primary diagnostic mean with a specificity, sensitivity and accuracy of around 95% [24,25] and with the potential to be used simultaneously for adequate treatment planning. In view of the improved options provided by intSE and other surgical techniques, factors such as the shape or mobility of the stones no longer appear to be of paramount importance. Instead, the focus has moved to factors related to the anatomy of the duct system and its variability, which may prevent the use of the new techniques. Differences involving the anatomy of the duct system have therefore also been taken into account through the introduction of the term “visible” [26]. It should also be mentioned that early treatment has been reported to be advantageous and associated with better outcomes. Patients should therefore be recommended to have treatment early after the diagnosis has been made [23].

Since 10–20% of stones are located intraparenchymally, they were not primarily accessible using TDS, any modification of intSE, or combined surgery, so that ESWL often remained as the only available treatment method. In addition to this, a further 5–15% of stones could not be successfully treated by any method. One report published in 2009 summarized the results after the treatment of around 4700 cases in five different salivary gland centers using minimally invasive treatment measures: 80.5% of all patients were completely cured (stone-free and symptom-free), 16.7% were partially cured (symptom-free, but not stone-free), and 2.9% had to undergo gland resection [16].

On the basis of these developments, treatment algorithms for sialolithiasis were published for centers that did not have regular access to ESWL [27] and those that did [26,28]. Although some differences were noted, all of the studies confirmed that the role of gland resection was becoming more and more limited [15,27,29]. The treatment algorithm published by our research group in 2009 included all of the known techniques and combinations of them [26], and a modification based on this was published by another group [28].

After this, further developments took place that refined and combined the successful techniques. Very good results were reported after the application of TDS [30,31,32,33,34,35,36] and sialendoscopy-assisted TDS [37,38,39,40,41]. Several groups reported transoral robot-assisted extraction of deeply located and large submandibular sialoliths [42,43,44], also in combination with sialendoscopic assistance [45,46]. A combined endoscopic–transcutaneous approach was also described for submandibular stones [39]. In another study, stones were removed with sialendoscopy and computed tomography (CT) navigation [47]. However, as these techniques are associated with considerable effort and costs, they may be reserved for special cases and do not appear to be first choice in routine treatment. In addition, most stones treated using the modifications mentioned above can also be treated alternatively with ESWL (if not accessible with sialendoscopy) or better with ISWL (if accessible with sialendoscopy).

ESWL continued to be included in treatment regimens after the improvement of techniques and standardization of ultrasound-guided application methods [48,49,50,51,52]. Combining ESWL with the known techniques, particularly intSE and TDS, was found to improve results by over 30% in one study of both major salivary glands [53] and by 5–10% in submandibular glands (SMGs) and over 20% in parotid glands (PGs) in another study [51].

Combined approaches, particularly in parotid sialolithiasis, were included in the algorithm and led to further remarkable developments. Results with combined approaches were published and indicated that ESWL may be partly replaced by a combination of transoral duct incision and intSE [54,55,56,57], which was a new approach for centers in which ESWL was not available. Other research groups reported experience in using a combination of a transcervical–transcutaneous approach and intSE, with good results in the parotid gland [30,55,56,57,58,59,60,61,62,63,64,65,66]. It was reported that the indication could be extended using image-guided navigation with ultrasound (US) [67], US in combination with a dye [68], and CT scanning [47,69]. Although a combined endoscopic–transcutaneous approach for submandibular stones was also described in one study [39], this technique did not gain such importance in this gland.

For nearly all of these combined approaches, sialendoscopy-guided intraductal shock-wave lithotripsy (ISWL) emerged as an alternative treatment modality during the last 5–10 years. ISWL is not included in any of the published algorithms [26], meta-analyses, reviews, or comprehensive studies [5,16,21,22,28,56,70,71,72].

Although the development of smaller, better adapted sialendoscopes led to a remarkable improvement in the options available with interventional sialendoscopy [11,12,13,14,73,74,75,76,77,78], ISWL did not become particularly important at the time and was not systematically investigated until the first decade of the 2000s. Several techniques were used in some studies, and only a few studies reported results for only one technique. When pneumatic ISWL techniques such as electrohydraulic or electrokinetic ISWL [79,80,81,82,83,84] were used, the results did not attract much attention, due to the risk of complications or side effects. Earlier publications assessed the effectiveness of various laser devices, but the results were in the range of 40–80% and did not suggest significant improvement [11,12,85,86,87,88,89,90,91,92,93,94].

Further development of devices and instruments suitable for ISWL, with high success rates, has taken place during the last 5–10 years. Intraductal pneumatic lithotripsy (IPL) and laser lithotripsy have emerged as the major and most effective ISWL techniques. Although good results were reported with a thulium laser [95] and in early publications with the use of excimer [85] or pulsed-dye lasers [86], these proved to be impractical and/or were too expensive. In addition, it is not clear whether difficult sialolithiasis or stones in difficult locations were included in the latter studies [85,86].

Among the lasers currently available, the Ho:YAG laser has been reported to be the most suitable [96], a finding that was underlined by success rates >80% in recent publications [97,98,99,100,101,102,103,104].

Pneumatic lithotripsy was applied and investigated in three studies, with good results of over 80% or 90% being reported [105,106,107]. In another study, IPL and intraductal laser lithotripsy (ILL) were compared in patients with submandibular gland sialolithiasis, both showing success rates > 90% [108].

The primary indication for ISWL is with stones in which gland-preserving treatment is not possible using the well-established techniques. All stones that are accessible with the sialendoscope but not treatable with conventional intSE in the parotid gland, and all stones that cannot be treated with conventional intSE or TDS in the submandibular gland, are recognized indications for ISWL [97,98,99,100,101,103,104,105,106,107].

ISWL has been used for difficult sialolithiasis as a single-mode treatment, and also in combination with other techniques. Difficult sialolithiasis is faced (a) when there is difficult anatomy in the ductal system (narrow duct, duct variations); (b) when the location is difficult (with poor accessibility); or (c) in the presence of multiple stones.

Since ISWL requires the insertion of interventional sialendoscopes with a working channel diameter of at least 0.4 mm for ILL and 0.65 mm for IPL [97,98,99,100,101,103,104,105,106,107], the options for performing ISWL are substantially dependent on the anatomical preconditions in the duct system. If the duct system is too narrow or there are duct variations, it may prohibit successful ISWL. Depending on the location of the stone, therefore, a transoral sialendoscopy-assisted approach, a transcutaneous sialendoscopy-assisted approach, or ESWL are the alternatives. 

Although ISWL may replace ESWL in some indications, particularly with parotid stones, ESWL has a place in treatment regimens if it is available. ESWL has proved to be of value in combination with other treatment modalities, particularly in difficult sialolithiasis. In some earlier studies, a combination of ESWL with conventional intSE, TDS, or other combined surgical techniques, all non-ISWL techniques, improved the results in PGs in 25–35% of cases and in SMGs in around 5% [51,53]. Sequential or simultaneous combination of ESWL and ISWL has been reported in a few studies, in which ISWL contributed to the improvement of treatment results and supplemented the combined treatment options. In an earlier study, Katz et al. reported that a combination with sequential use of the two techniques led to an improvement in the results of around 30% [10]. Koch et al. reported that patients presenting with difficult sialolithiasis could be treated with ISWL after the application of ESWL with a success rate of > 90%. In that study, stones that were not adequately visible and/or accessible for intSE could be mobilized using ESWL and were ultimately successfully treated with ISWL in 95% of cases in both major salivary glands [109]. A combination of ISWL and TDS has also been reported in cases of difficult sialolithiasis in the SMG. Stones that are not removable with TDS (persistent sialolithiasis), residual stones in patients with multiple sialolithiasis, and recurrent stones after TDS in posthilar to intraparenchymal locations are important new indications for ISWL, as has been shown in recent publications [103,104,105,107,110]. The topic was addressed specifically in one of these papers, in which persistent, residual, and recurrent stones after TDS were treated with ISWL, with a success rate of 97% [110].

Simultaneous application of ultrasound and sialendoscopy has been reported to further improve the treatment options in selected cases, particularly in parotid sialolithiasis [111,112]. US-guided stone extraction alone, without sialendoscopy, has been described [113], but appears to play only a minor role, if any, if sialendoscopy is not available.

Various comprehensive reports [5,16,21,22], reviews [70], and meta-analyses [71] have been published since 2010, but the potential impact of ISWL has not been addressed in any of the treatment strategies. Several treatment algorithms have been proposed, highlighting the decline in the importance of ESWL and the increase in the importance of combined techniques. However, the growing impact of ISWL and new options for combining it with familiar treatment modalities have not been considered, or at least not adequately [28,56,72], with the exception of one recent review [1]. Although it is clear that ESWL is declining in importance, it may perhaps be underrepresented.

Taking all of these changes into account, we now consider that the treatment algorithms we published in 2009 [26] should be updated. All relevant techniques that contribute to rapid, effective, and cost-effective management of sialolithiasis without excessive effort have been included. Prognostic factors such as the size, location, and mobility of stones have been included [23]. However, the shape of stones does not appear to play any important role in the era of ISWL and has not been taken into consideration. Due to the options available with ISWL and further developments in combined surgical techniques, it was considered that the role of factors relating to the anatomy of the ductal system is more adequately covered by replacing the term “visible” with the term “accessible.” The use of this term is intended to emphasize the fact that, in view of recent developments—particularly concerning ISWL—the accessibility of a stone in addition to its size is of paramount importance in comparison with all of the other prognostic factors mentioned in the literature.

**Figure 1:**
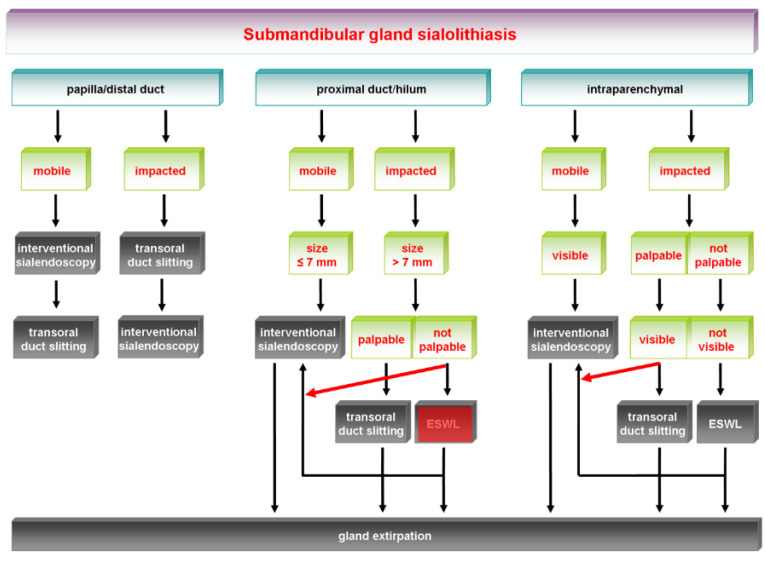
Submandibular gland sialolithiasis: earlier treatment algorithms [26], with changes indicated (red-marked boxes, red arrows). ESWL, extracorporeal shock-wave lithotripsy.

**Figure 2:**
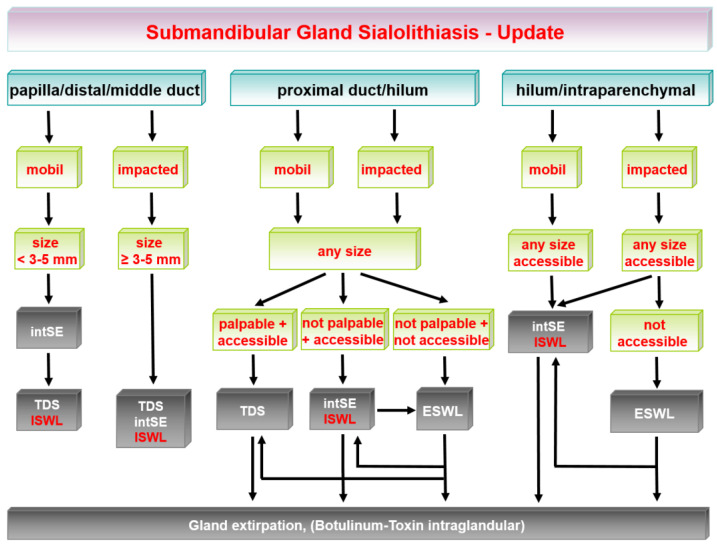
Submandibular gland sialolithiasis: current/updated treatment algorithms. ESWL, extracorporeal shock-wave lithotripsy; intSE, interventional sialendoscopy; ISWL, intraductal shock-wave lithotripsy; TDS, transoral.

**Figure 3:**
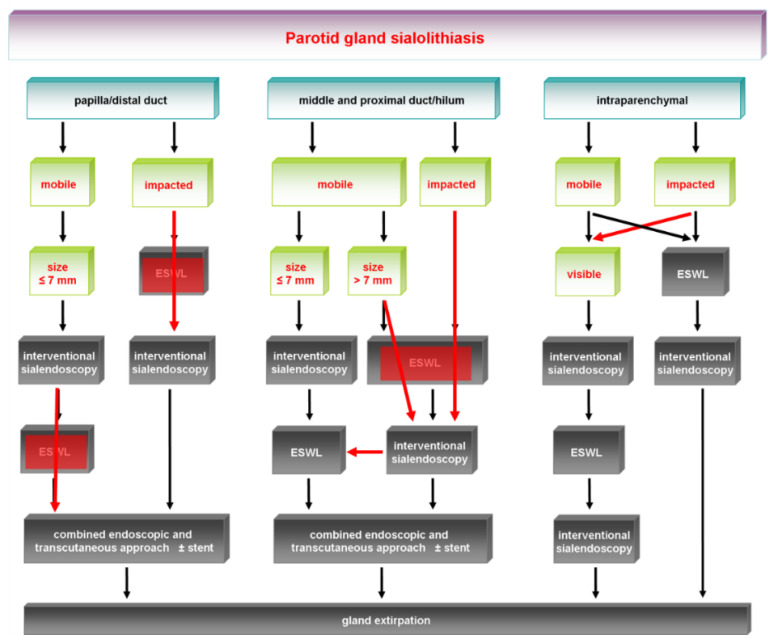
Parotid gland sialolithiasis: earlier treatment algorithms [26], with changes indicated (red-marked boxes, red arrows). ESWL, extracorporeal shock-wave lithotripsy.

**Figure 4:**
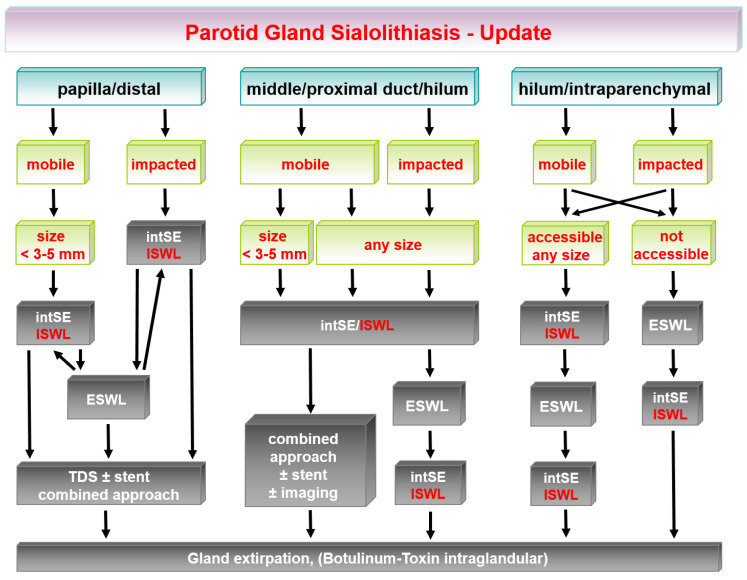
Parotid gland sialolithiasis: current/updated treatment algorithms. ESWL, extracorporeal shock-wave lithotripsy; intSE, interventional sialendoscopy; ISWL, intraductal shock-wave lithotripsy; TDS, transoral duct surgery.

The earlier algorithm of SMG sialolithiasis is shown in Figure 1 and its update in Figure 2. The earlier algorithm of PG sialolithaisis is depicted in Figure 3 and its update in Figure 4. Changes in the impact of treatment modalities (e.g., ESWL) and alterations in treatment strategies are marked with red boxes and/or arrows. The following sections provide more detailed analysis of the ways in which recent developments have influenced the therapeutic strategy in sialolithiasis for each of the major salivary glands.

## 2. Updated Treatment Algorithm for Submandibular Gland Stones

Conservative measures (gland massage during or after gland stimulation), including instrumental dilation of the papilla, are always primarily indicated. Very small stones may then be spontaneously washed out.

### 2.1. Stones at the Papilla and in the Distal and Middle Duct

Small mobile stones (≤3–5 mm) are primarily an indication for basket or forceps-controlled extraction with intSE. As the ostium of the papilla is narrow, a (mini-) papillotomy is necessary to remove the stone in the great majority of cases. Since intSE is possible in only 5–10% of cases, TDS is usually the first choice. Due to the excellent accessibility of the duct system, stones from the papilla to the middle duct system can be extracted independently of their size or state of impaction. Mechanical fragmentation or ISWL may be indicated in patients with unfavorable anatomic conditions (e.g., reduced mouth opening or even trismus, small and narrow mandibular arch, deep floor of the mouth, extensive gagging reflex) or in those who decline TDS in order to avoid general anesthesia. In some of these cases, mechanical fragmentation with microdrills may be an alternative if the stones have a smooth consistency and are not too large. If mechanical fragmentation or ISWL is performed, papillotomy or a retropapillary duct incision may be necessary in order to insert sialendoscopes with larger diameters. ESWL is not indicated for stones in this location.

### 2.2. Stones in the Proximal to Hilar Duct System

Small mobile stones (≤3–5 mm) that can be visualized and are adequately accessible during sialendoscopy may be retrieved with intSE. However, this is hardly ever possible, due to the curved and very narrow duct system. ISWL is a valuable alternative in such cases. Immobile, impacted larger stones (<10 mm) are an indication for TDS, but can be also be removed with mechanical fragmentation or ISWL followed by fragment extraction. ISWL in particular may be preferable in larger and impacted stones in order to reduce surgical trauma, since only a limited incision is necessary (see above). Extended TDS/sialendoscopy-assisted TDS is the first choice in large stones (>10 mm), as it is an effective and fast modality. It can be performed if the stone can be visualized during sialendoscopy, is not located too far within the duct system (depth measured with the sialendoscope <6.5–7.0 cm), and is palpable. ISWL may be indicated in difficult cases when the patient has unfavorable anatomic conditions, in order to avoid general anesthesia (see above). When the stones are accessible, ESWL may be offered only in selected cases—e.g., if the patient declines all other treatment modalities or in difficult sialolithiasis. ESWL may then be performed with the main intention of fragmenting and mobilizing less good accessible or inaccessible stones so that they become amenable again for intSE or ISWL, to retrieve the fragments. In these cases, typically representing difficult sialolithiasis, combined treatment is often necessary to ensure success.

### 2.3. Posthilar to Intraparenchymal Stones

Only approximately 10% of all stones are in an intraparenchymal location. Conservative measures may be sufficient for asymptomatic stones.

If the calculi can be visualized during endoscopy, an attempt may also be made to extract small, mobile stones (<3–5 mm) using intSE, including mechanical fragmentation or ISWL, which is more effective than mechanical fragmentation with intSE. However, due to the specific anatomy of the duct system, with poor accessibility, this is possible only in a minority of cases. The indication for ISWL is heavily dependent on the anatomical situation, which determines the visibility and accessibility of the stones.

Extended TDS with opening of the gland (submandibulotomy) is indicated for large stones (>10 mm) that are palpable from inside the mouth. If stones cannot be visualized endoscopically, are not palpable, and are located too far inside the duct system (>6.5–7.0 cm), ESWL is indicated. If ESWL is performed as a single treatment modality, its success rate is significantly reduced, particularly for stones >10 mm in size. However, ESWL can be supplemented with or later combined with ISWL or TDS after adequate mobilization.

In summary, the main change in the treatment algorithm is that the development of ISWL has led to a reduction in the indications for ESWL. Experience has also shown that ISWL can replace TDS, particularly with smaller stones. Nevertheless, TDS is still the most important treatment modality, due to the good accessibility of the duct system. Although ESWL has become less important, it is still a substantial element in the treatment algorithms, particularly for intraparenchymal stones that are not visible or accessible.

Removal of the gland should be considered in therapy-resistant cases, when there are persistent symptoms, and in accordance with the patient’s wishes. If a patient is inoperable for any reason, injection of botulinum toxin may be considered. Multiple sialolithiasis, even in intraparenchymal locations, is not in itself an indication for gland resection, as combined treatment provides good results. However, due to the increased treatment effort involved, the patient needs to receive adequate counseling. 

Figure 1 shows the earlier algorithm for the management of submandibular stones published in 2009. The updated algorithm is shown in Figure 2. The changes are highlighted with red boxes and arrows.

## 3. Updated Treatment Algorithm for Parotid Gland Stones

Conservative measures (gland massage during or after gland stimulation), including instrumental dilation of the papilla, are always primarily indicated. Very small stones may then be spontaneously washed out.

### 3.1. Stones in the Papilla and Distal Excretory Duct

Mobile stones not exceeding 3–5 mm in size are an ideal indication for extraction with intSE using instruments such as a basket or forceps, if necessary after application of ISWL. Endoscopic mobilization or fragmentation is indicated for stones 5–7 mm in size. If the stones have a weak consistency, mechanical fragmentation using microdrills is a possible solution in some cases. However, in general, in particular if the stones are of a hard consistency and/or are greater than 7 mm, ISWL represents an excellent treatment modality with high success rates. Major changes from the earlier algorithm are the fact that intSE or ISWL are the first choice if the anatomy of the duct system permits. If the anatomy is unsuitable or ISWL is not available, a combined retropapillary transoral approach, if necessary combined with intSE with or without stent implantation, is another reasonable solution. Combined transcutaneous–endoscopically guided stone retrieval is another modality in this location. ESWL is no longer a preferred treatment modality and is indicated only in selected cases—e.g., when there is unfavorable duct anatomy and/or a difficult stone location (e.g., extraductal). ESWL can then again be supplemented with intSE and ISWL.

TDS methods or other major surgical manipulations at the papilla are obsolete as a primary therapeutic measure, as there is a substantial risk for stenosis developing. However, a minipapillotomy may be carried out without the risk of creating a stenosis with stones/fragments that have been grasped by various instruments during interventional sialendoscopy but have proved to be too large to pass through the papilla. A minipapillotomy consists of a superficial incision in the papilla, not involving the duct epithelium and extending to a maximum of 3–4 mm. When these rules were followed, no complications were observed in our cases. If distal stones are present in multiple sialolithiasis, ESWL may play a more important role as part of a combined treatment.

### 3.2. Stones in the Middle or Proximal Duct and Hilar Region

Small, mobile stones (≤3–5 mm) are primarily an indication for intSE or ISWL, independently of their size or impaction. If intSE or ISWL are not feasible (mainly because the stone is not accessible or visible), ESWL is indicated if available, and it can then again be supplemented with intSE or ISWL.

If the anatomy of the duct system is unsuitable—e.g., due to narrowness of the duct lumen or duct variations—then ESWL, intSE, or ISWL may not be promising, leaving the combined endoscopic–transcutaneous approach as an alternative. The successful application of the latter also depends on the visibility of the stone with the sialendoscope, or at least on the accessibility with an instrument inserted into its working channel. The modifications of the combined approaches described (US with or without transcutaneous stone extraction) are further approaches belonging to this category.

Recent publications have reported that simultaneous application of ultrasound and sialendoscopy can further improve the treatment options in difficult situations [111,112]. When the various treatment modalities are considered, a declining trend in the indications for ESWL is evident, particularly in stones located up to the proximal duct. However, if there is difficult duct anatomy and/or an extraductal stone location, ESWL may be the only reasonable option, as the competing modalities are not effective. However, it should also be mentioned that certain contraindications prevent the use of ESWL (e.g., a cardiac pacemaker).

### 3.3. Hilar to Intraparenchymal Stones

Up to 20% of all stones are in an intraparenchymal location. In general, these stones are more commonly impacted. Conservative measures may be sufficient for asymptomatic stones.

If the calculi can be visualized during endoscopy, an attempt may also be made to extract small, mobile stones using endoscopy, mobilization, or fragmentation (see above). IntSE and ISWL may be applied in favorable anatomic situations. As the stones are often not visible and/or accessible with the sialendoscope, ESWL is a valuable and sometimes the only treatment modality if gland preservation is intended. ESWL is then conducted to achieve stone fragmentation and mobilization of stones or fragments. After adequate mobilization, ESWL may be supplemented by intSE or combined with ISWL or even a combined approach, if a stone is accessible.

Patients with treatment-resistant stones and those in whom ESWL is contraindicated have an indication for the combined endoscopic–transcutaneous approach. The prerequisite for this is endoscopic accessibility of the stone. The combined transcutaneous–endoscopic approach has a limited indication for stones in this location. If it is indicated, a higher failure rate may be expected. Successful therapy may be achieved if the procedure is performed with simultaneous navigation using imaging tools such as US [67,68] or CT [69]. In selected cases, ultrasound-navigated, sialendoscopy-guided stone or fragment extraction can lead to success [111]. 

If multiple sialolithiasis is present, a combination of ESWL and ISWL in particular is a promising step forward in efforts to preserve the glands in this situation as well.

Parotidectomy is indicated in any treatment-resistant sialolithiasis with symptoms and persistent inflammation despite successful therapy, and after counseling of the patient. If a patient is inoperable for any reason, injection of botulinum toxin may be considered.

In summary, experience shows that as ISWL has become more important, ESWL has lost its importance in the management of parotid stones, although it is still a substantial element in the treatment algorithms in units that have access to it. Maturing ISWL techniques represent a major advance in treatment for (difficult) sialolithiasis, and the gland resection rate may be further reduced. If several stones are present, the treatment, which is then often combined, also follows the algorithms described here.

Changes in the earlier treatment algorithm are shown in Figure 3, with major changes indicated and highlighted with red boxes and red arrows. The current algorithms are depicted in Figure 4.

## 4. Conclusions

In conclusion, recent advances in sialendoscopy-guided treatment for salivary stones have been made and have been reported in published studies during the last 5–10 years. The literature results point to the growing importance of ISWL, for which very good results have been reported. This has led to an expansion of the scope for less invasive, gland-preserving treatment. It is an effective, but not always a faster form of treatment. In both glands, and in PGs in particular, ESWL, combined or endoscopy-assisted surgery, and TDS methods may be partly replaced. ESWL is still of value, particularly in combination treatment, which further improves the results. ESWL can also make stones amenable to ISWL treatment, and the two methods can supplement each other. As combination treatment is more promising in cases of difficult sialolithiasis, there may be greater effort involved in such cases. This must be discussed with the patient, and adequate counseling is needed. As all of the procedures can be performed with local anesthesia, general anesthesia can be avoided in the great majority of cases, representing a major advantage for patients.

It may be argued that the increased effort involved in gland-preserving treatment is not justified and that resection of the gland can be performed quickly and without major complications in most cases. This may be partly justified, particularly for the SMG. However, unfavorable cosmetic aspects (cervical scar) and reduced saliva production are side effects that always occur. In this context it should be mentioned that a cervical scar can be avoided when the SMG is removed by a transoral, e.g., robotic assisted approach [114,115]. Other complications (collateral damage to the lingual nerve and the mandibular branch of the facial nerve) are rare and should be discussed with the patient preoperatively. With regard to parotidectomy, the situation is different. Paresis or permanent paralysis of the facial nerve in particular is rare. However, unfavorable cosmetic aspects (scarring and volume defects) may be significant and can only be relieved with a modified skin incision and additional measures (e.g., superficial musculoaponeurotic system flap and fat implantation). As the whole gland is usually removed, Frey’s syndrome may be more frequent and is perceived by patients as uncomfortable. Multiple additional measures have been investigated to prevent this, but without a single measure being reported to have a significant preventative effect. If the condition is present and the patient wishes treatment, Frey’s syndrome can be treated with an intracutaneous botulinum toxin injection. However, the need for repeated injections after performance of the Minor starch-iodine test every 3–6 months requires effort and compliance from the patients, and this is not always taken into account when gland-ablating surgery is discussed preoperatively. In the light of this, gland-preserving treatment should always be considered as the first choice.

## Data Availability

Not applicable.
